# Supplementary material for: Social and psychological adversity are associated with distinct mother and infant gut microbiome variations
Source: Nat Commun. 2023 Sep 20;14:5824. doi: 10.1038/s41467-023-41421-4 (PMC10509221; doi:10.1038/s41467-023-41421-4)
Supplement: Supplementary file 1 — Supplementary Information [file 41467_2023_41421_MOESM1_ESM.pdf]

**Supplementary Information**

# Social and psychological adversity are associated with distinct mother and infant gut microbiome variations

Barbara B. Warner[&*], Bruce A. Rosa[&], I. Malick Ndao, Phillip I. Tarr, J. Phillip Miller, Sarah K. England, Joan L. Luby, Cynthia E. Rogers, Carla Hall-Moore, Renay E. Bryant, Jacqueline D. Wang, Laura A. Linneman, Tara A. Smyser, Christopher D. Smyser, Deanna M. Barch, Gregory E. Miller, Edith Chen, John Martin and Makedonka Mitreva[*]

*** Correspondence:** Warner BB, warnerbb@wustl.edu; Mitreva M, mmitreva@wustl.edu

**& Equal contribution:** Warner BB, warnerbb@wustl.edu; Rosa BA, barosa@wustl.edu

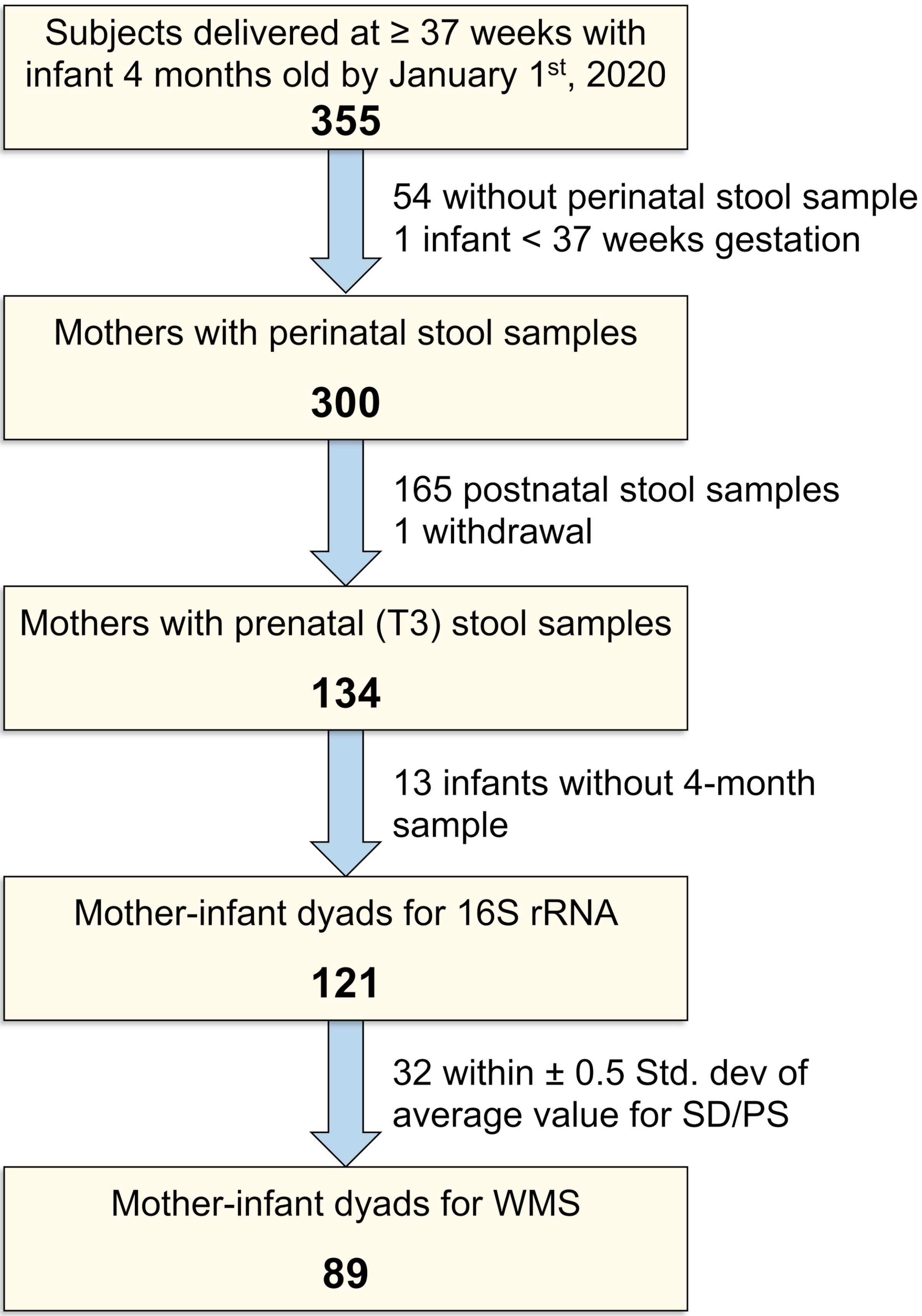


**Supplementary Fig. 1**. Flowchart summarizing the sample selection process from the 355 subjects in the larger study cohort, to the 121 mother-infant dyads used for the 16S rRNA analysis and the 89 dyads used for the WMS analysis.

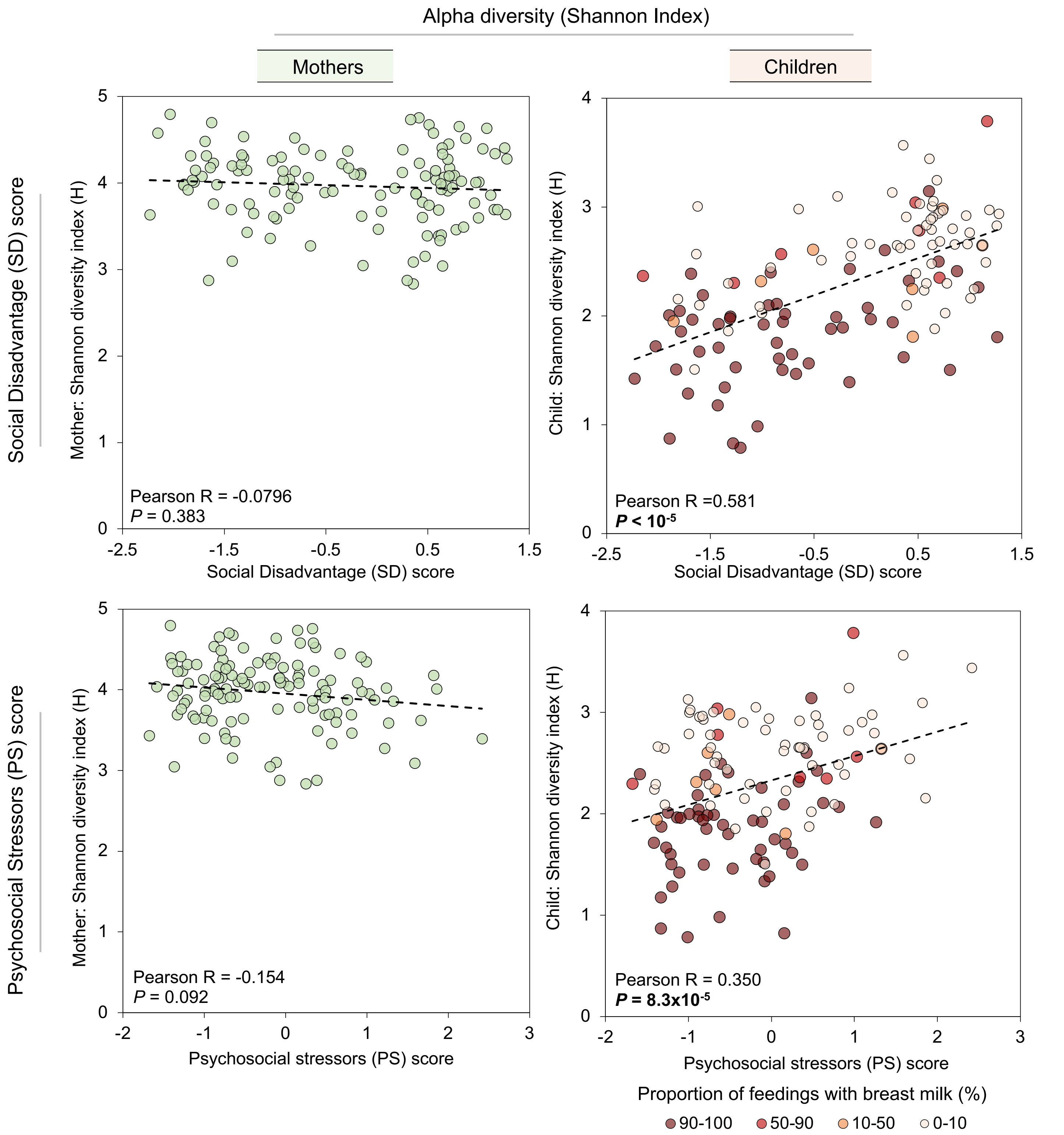


**Supplementary Fig. 2**. GM sample diversity comparisons with Social Disadvantage (SD) and Psychosocial Stressors (PS) scores. SD scores and PD scores do not significantly correlate with GM α-diversity (Shannon diversity index) in the 121 GM samples from the mothers. Two-sided T-distribution correlation tests were used for all comparisons (no adjustment for multiple comparisons).

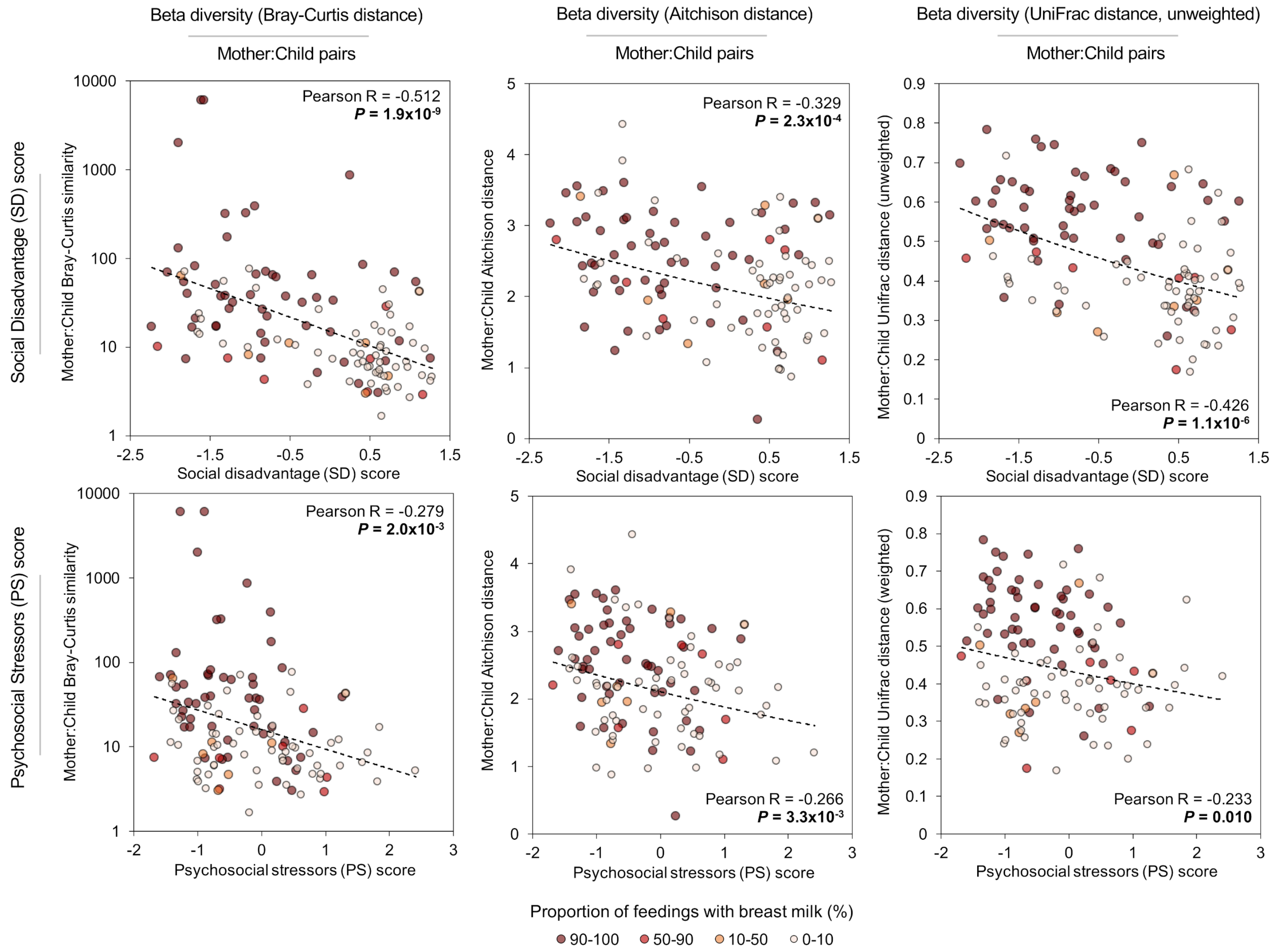


**Supplementary Fig. 3**: Additional β-diversity metrics including Bray-Curtis dissimilarity, Aitchison distance and unweighted UniFrac distance, all show significant negative correlations between β-diversity and both social disadvantage and psychological stressors, using two-sided T-distribution correlation tests (no adjustment for multiple comparisons).

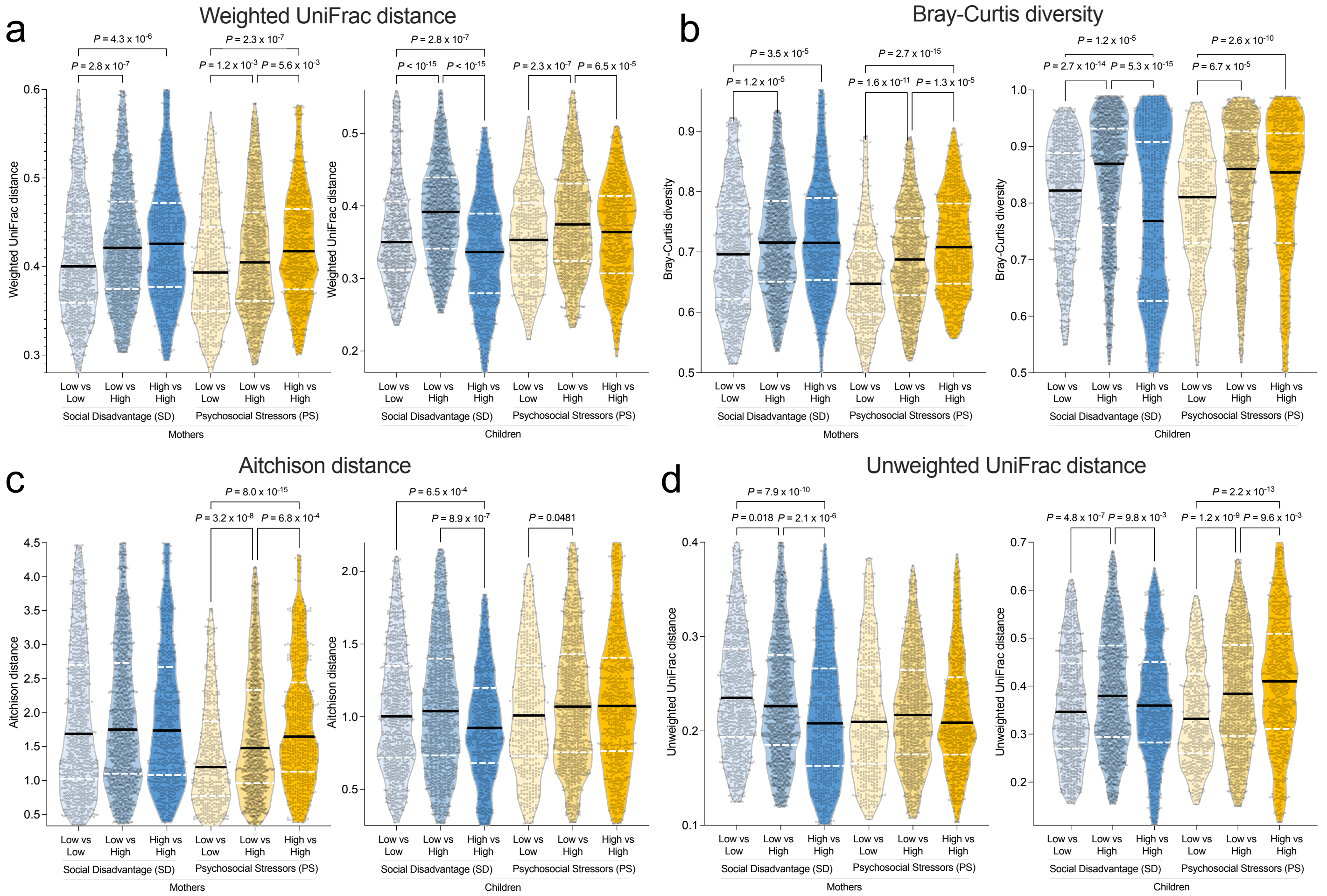


**Supplementary Fig. 4.** Comparisons of β-diversity between sample sets based on high-vs-low SD and PS, for mothers and children quantified by (a) Weighted UniFrac distance, (b) Bray-Curtis diversity, (c) Aitchison distance and (d) Unweighted UniFrac distance. Two-sided FDR-corrected Mann Whitney U-tests were used to test significance. On the violin plots, thick black lines indicate the median values, white dashed lines indicate the quartiles of the data range, the width of the shaded areas represent the proportion of data points located at the given weighted UniFrac distance, and the height of the shaded areas spans from the minimum to the maximum value. The number of values used for the tests were the number of unique biologically independent sample pairs in each group (n=903 low-SD vs low-SD, n=1505 high-SD vs low-SD, n=595 high-SD vs high-SD, n=496 low-PS vs low-PS, n=1152 high-PS vs low-PS, n=630 high-PS vs high-PS).

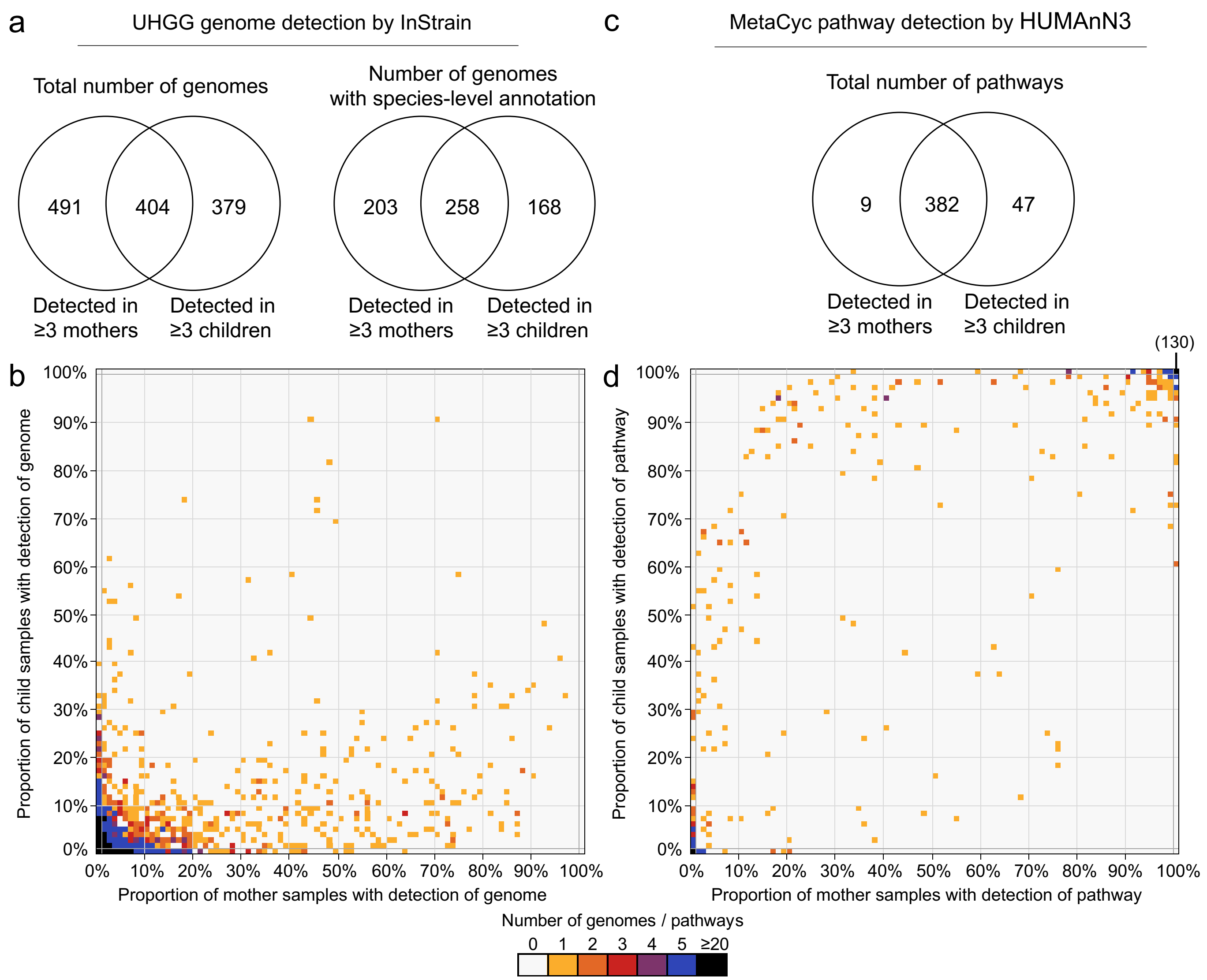


**Supplementary Fig. 5**. Genome and pathway detection in the 89 mother and 89 child WMS samples. (a) The total number of bacterial genomes and genomes with species-level taxonomic annotation detected in at least 3 maternal samples and/or at least 3 child samples. (b) For each of the 2,219 bacterial genomes detected in any sample, the proportion of mother and child samples that were detected in the dataset. (c) The total number of metabolic pathways detected in the GM from at least 3 mother and at least 3 child stools. (d) For each the 468 metabolic pathways detected in any sample, the proportion of mother and child samples with detection in the dataset.

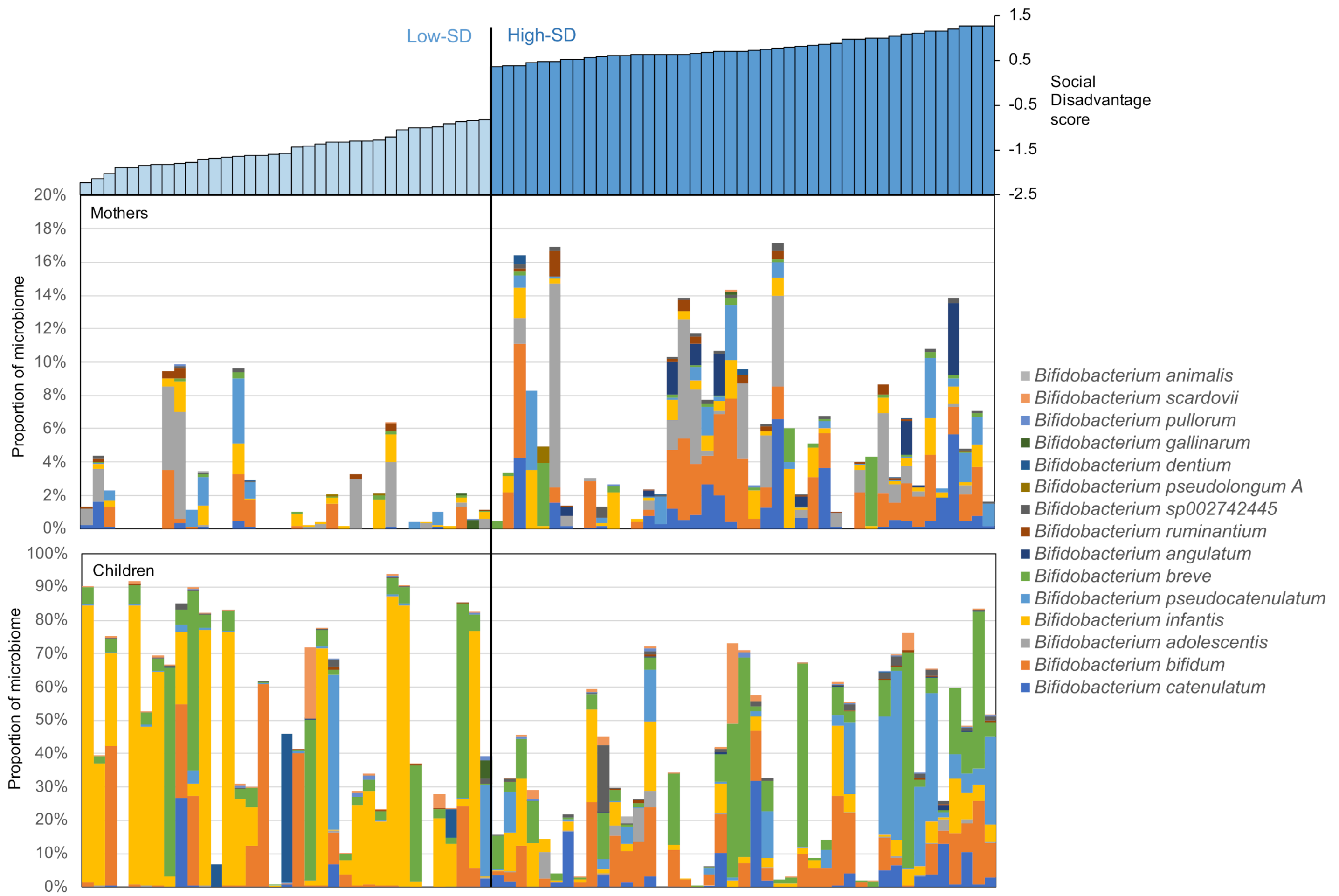


**Supplementary Fig. 6.** The relative abundance of *Bifidobacterium* species identified in all mothers and children in the high-SD (n=42) vs low-SD (n=35) comparisons.

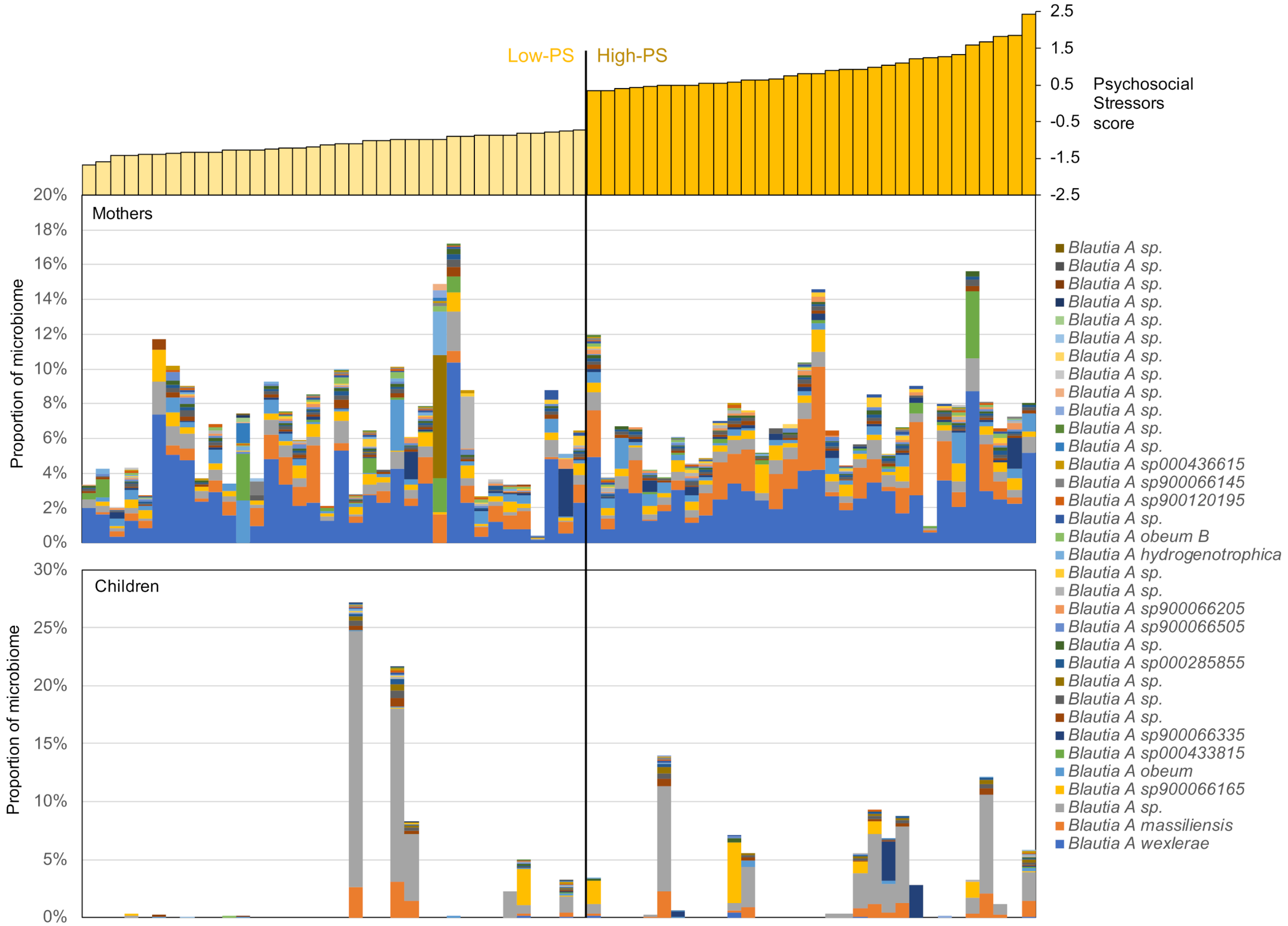


**Supplementary Fig. 7.** The relative abundance of *Blautia* species identified in all mothers and children in the high-PS (n=32) vs low-PS (n=36) comparisons.

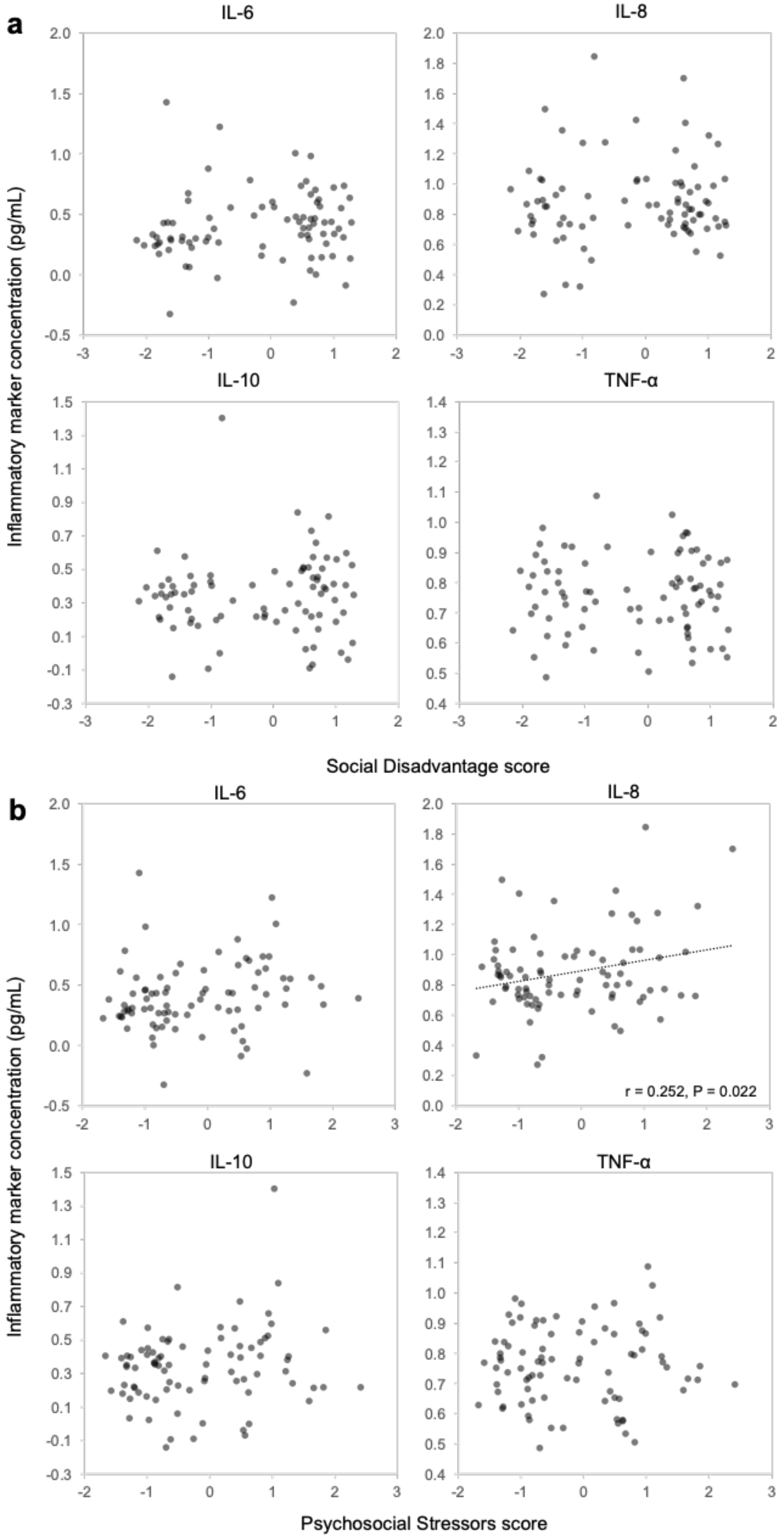


**Supplementary Fig. 8.** Comparison of (a) Social Disadvantage scores and (b) Psychosocial Stressors scores to maternal third trimester inflammatory marker serum concentrations. Only IL-8 and PS scores correlated significantly. All tests were performed using two-sided T-distribution correlation tests (no adjustment for multiple comparisons, n=83, degrees of freedom = 81). Detailed correlation statistics are shown in Supplementary Data 1f.

## Supplementary Tables

**Supplementary Table 1** Random Forest (RF) machine learning predictive accuracy for high-vs-low inflammatory markers based on GM taxonomic and pathway profiles. Prediction accuracy was consistently greater for IL-6 than for the other inflammatory markers. Bolded values correspond to comparisons for which P < 0.005 (Binomial distribution tests, with FDR correction).

| | | Prediction accuracy | | | | FDR-adjusted P value for prediction accuracy | | | |
|---|---|---|---|---|---|---|---|---|---|
| | | IL-6 | IL-8 | IL-10 | TNF-α | IL-6 | IL-8 | IL-10 | TNF-α |
| Mothers | Genomes | 48.9% | 40.0% | 43.1% | 58.2% | 0.747 | 0.940 | 0.913 | 0.590 |
| | Pathways | 51.1% | 44.0% | 51.0% | 56.4% | 0.597 | 0.889 | 0.597 | 0.597 |
| Children | Genomes | 66.7% | 60.0% | 47.1% | 56.4% | 0.029 | 0.202 | 0.796 | 0.672 |
| | Pathways | 64.4% | 54.0% | 49.0% | 47.3% | 0.102 | 0.597 | 0.667 | 0.889 |
