# Supplementary material for: Social and psychological adversity are associated with distinct mother and infant gut microbiome variations
Source: Nat Commun. 2023 Sep 20;14:5824. doi: 10.1038/s41467-023-41421-4 (PMC10509221; doi:10.1038/s41467-023-41421-4)
Supplement: Supplementary file 3 — Description of additional supplementary files [file 41467_2023_41421_MOESM3_ESM.pdf]

Description of Additional Supplementary files

Supplementary Data 1: Detailed statistical output and results from all statistical tests. FDR-adjustment is indicated when performed, and two-tailed tests are used in all instances when applicable. (a) Complete patient characteristics at study entry for each of the primary comparisons of interest. "Low" and "High" Disadvantage and Psych scores are separated according to the distribution of the metadata, as shown in Figure 1. (b) Shapiro-Wilk tests for Normality. P values ≤ 0.05 are considered to not be normally distributed, and non-parametric tests are used for comparisons of those variables. (c) Two-tailed T-tests for significant differences for normally-distributed data (unequal variance). (d) Chi-square test results for significant differences in categorical data. (e) Two-sided Mann-Whitney U / Wilcoxon Rank Sum tests for significant differences for normally-distributed data. (f) Pearson correlation analysis to identify significant associations between metadata, using a two-tailed T distribution test. (g) Statistics to identify the optimal number of clusters for clustering the 16S rRNA samples (n = 121 for children and mothers). Bolded values indicate the optimal statistic / number of clusters for each sample set. (h) Binomial distribution test (cumulative) to determine significance of Random Forest prediction compared to random selection. (i) Differential abundance statistics for each UHGG genome in each comparison. Average relative abundance values are based on values from Supplementary Data 2. MDA = Mean decrease in accuracy (%) of the RF model. LEfSe Kruskal Wallis test and ANCOM-BC2 P values were FDR-corrected for the top 25 taxa for Figures 6 and 7. (j) Differential abundance statistics for each MetaCyc pathway in each comparison. Average relative abundance values are based on values from Supplementary Data 2. MDA = Mean decrease in accuracy (%) of the RF model. LEfSe Kruskal Wallis test and ANCOM-BC2 P values were FDR-corrected for the top 25 taxa for Table 2.

Supplementary Data 2: Database of sample data, including metadata, indications of sample groups for each comparison, relative 16S ASV abundance, relative MGS genome abundance and relative MGS pathway abundance values.

Supplementary Data 3: Full ASV sequences for each unique ASV identifier from Supplementary Data 2.
