# Supplementary material for: Social and psychological adversity are associated with distinct mother and infant gut microbiome variations
Source: Nat Commun. 2023 Sep 20;14:5824. doi: 10.1038/s41467-023-41421-4 (PMC10509221; doi:10.1038/s41467-023-41421-4)
Supplement: Supplementary file 7 — Reporting Summary [file 41467_2023_41421_MOESM7_ESM.pdf]

nature portfolio

Corresponding author(s): Makedonka Mitreva

Last updated by author(s): 07-02-2023

Reporting Summary

Nature Portfolio wishes to improve the reproducibility of the work that we publish. This form provides structure for consistency and transparency in reporting. For further information on Nature Portfolio policies, see our Editorial Policies and the Editorial Policy Checklist.

Statistics

For all statistical analyses, confirm that the following items are present in the figure legend, table legend, main text, or Methods section.

n/a	Confirmed	
☐	☒	The exact sample size (*n*) for each experimental group/condition, given as a discrete number and unit of measurement
☐	☒	A statement on whether measurements were taken from distinct samples or whether the same sample was measured repeatedly
☐	☒	The statistical test(s) used AND whether they are one- or two-sided<br>*Only common tests should be described solely by name; describe more complex techniques in the Methods section.*
☐	☒	A description of all covariates tested
☐	☒	A description of any assumptions or corrections, such as tests of normality and adjustment for multiple comparisons
☐	☒	A full description of the statistical parameters including central tendency (e.g. means) or other basic estimates (e.g. regression coefficient) AND variation (e.g. standard deviation) or associated estimates of uncertainty (e.g. confidence intervals)
☐	☒	For null hypothesis testing, the test statistic (e.g. *F*, *t*, *r*) with confidence intervals, effect sizes, degrees of freedom and *P* value noted<br>*Give P values as exact values whenever suitable.*
☒	☐	For Bayesian analysis, information on the choice of priors and Markov chain Monte Carlo settings
☒	☐	For hierarchical and complex designs, identification of the appropriate level for tests and full reporting of outcomes
☐	☒	Estimates of effect sizes (e.g. Cohen's *d*, Pearson's *r*), indicating how they were calculated

Our web collection on statistics for biologists contains articles on many of the points above.

Software and code

Policy information about availability of computer code

Data collection

Study Design, clinical cohort, and ethics statement: Mother-child dyads were drawn from a larger prospective observational study, the Early Life Adversity Biological Embedding and Risk for Developmental Precursors of Mental Disorders Study (eLABE), with pregnant women (N=395) recruited from the March of Dimes (MOD) Prematurity Research Center at Washington University in St. Louis (USA) between 2017-2020 and delivering at Barnes Jewish Hospital in St. Louis. Within the MOD cohort perinatal (T3, at delivery or postnatal) stool samples were collected, with only T3 samples eligible for this study (N=134 of 300 perinatal samples), 121 of which also had corresponding 4-month infant stool samples. At each trimester of pregnancy, measures of maternal depression, experiences of stress, as well as demographic and clinical information including insurance, education, address, household composition, pre-pregnancy BMI, and route of delivery were obtained from participants or extracted from the medical record by trained staff.
The latent factor of maternal social disadvantage (SD) is calculated using: Insurance status verified in the 3rd trimester from the medical record and maternal self report; Income to Needs Ratio in each trimester based on self-reported family income and houshold size (1.0 being the poverty line for the U.S.); highest self-reported maternal educational level; Area Deprivation Index, a national multidimensional geotracking method based on census block data providing percentile rankings of neighborhood disadvantage status; and maternal nutrition over the past year categorized using the validated Healthy Eating Index (using National Cancer Institute. The Healthy Eating Index – Population Ratio Method. Updated December 14, 2021) obtained using the Diet History Questionnaire (DHQII).
The latent factor of maternal psychosocial stressors (PS) is calculated using: The Edinburgh Postnatal Depression Scale (EPDS) completed in each trimester; Perceived Stress scale (PSS) completed in each trimester and averaged over trimesters; and a one-time lifetime STRAIN survey, a comprehensive measure of lifetime stressful and traumatic life events. Experiences of discrimination based on race were assessed using the Everyday Discrimination Scale.

For infants, gestational age was determined by the best obstetric estimate using last menstrual period or earliest ultrasound dating. Birthweight and route of delivery were extracted from the electronic medical record delivery note. Breastfeeding data were collected by

parental report at the time of home stool sample collection and based on the Center for Disease Control Infant Feeding Practices II study food frequency checklist data.
Maternal serum samples were collected in each trimester, processed within 12 hours of collection, and stored at -80°C. Spontaneously generated stool (feces) from mothers and infants were collected from home using a community-based courier system available 24 hours per day and stored at -80°C as previously described. All samples were processed in the laboratory of Dr. Phillip Tarr, where DNA was extracted from stools that had been frozen at -80°C since acquisition using the Qiagen (Hilden, Germany) QIAamp Power Fecal Pro DNA Kit (catalogue #51804), and the automated QIAcube99 (Qiagen). Briefly, 100 mg of stool was suspended in 1.2 mL of stool lysis buffer in a 2 mL screw cap tube containing a mix of 6-8 zirconium beads (2.3 mm, RPI Corporation, Mount Prospect, IL) and 0·1 mL of acid washed glass beads (0.4-0.6 mm, Sigma, MO). This suspension was homogenized by bead beating (FastPrep 24, MP Biomedical, Santa Ana, CA) (6.0 set point, 2 minutes), and centrifuged (14,000 x g, 3 minutes, room temperature). 350 µL of clear supernatant was then loaded onto the QIACube rotor adaptor for automated DNA purification. The supernatant was then treated on board with Inhibitor Removal Technology (IRT(Qiagen)), to remove inhibitors of subsequent enzymatic. DNA was eluted in 200 µL volume.

16S sequencing data: DNA extracted from stool was sequenced on an Illumina MiSeq, producing 2x250bp paired-end reads spanning the V4 hypervariable region for 242 samples (121 samples from mothers and 121 from their matched children). 16S sequencing was performed by the Genome Technology Access Center at McDonnell Genome Institute (GTAC@MGI) at Washington University in St. Louis School of Medicine, USA. Primer sequences used were forward primer (5’-AATGATACGGCGACCACCGAGATCTACACATCGTACGTCGTCGGCAGCGTCAGATGTGTATAAGAGACAGANNNNNGTGCCAGCMGCCGCGGTAA-3’) and reverse primer (5’-CAAGCAGAAGACGGCATACGAGATACCTACTGGTCTCGTGGGCTCGGAGATGTGTATAAGAGACAGNNANNNGGACTACHVGGGTWTCTAAT-3’).

Whole metagenome shotgun (WMS) sequencing data: For the WMS sequence analysis, samples were divided into “high” and “low” SD and PS based on the distribution of these values across the sample set. Samples above the average value +0.5 standard deviations were considered “high” and samples below the average value -0.5 standard deviations were considered “low” (35 “low-SD”, 43 “high-SD, 36 “low-PS” and 32 “high-PS”). After this selection, whole Metagenome Shotgun (WMS) datasets for were generated for 178 of the 242 samples (89 samples from mothers and 89 from their respective children) on the Illumina NovaSeq S4 (150bp paired end reads). WMS sequencing was performed by the Genome Technology Access Center at McDonnell Genome Institute (GTAC@MGI) at Washington University in St. Louis School of Medicine, USA. For each sample, ~6Gbp was generated, producing between 2.7 and 237.2 million reads per sample (average 61.5 million reads).

Data analysis

16S samples:
Data were imported into QIIME2 using standard methods and the developer’s docker container (qiime2/core:2018.8). V4 region amplicons were assembled and denoised using the QIIME2 method ‘DADA2 denoise-paired’. Processed V4 amplicons were grouped into amplicon sequence variants (ASVs) with 100% sequence similarity. Two reagent-only samples per plate were included to indicate the potential degree of contamination in the absence of bacteria in the samples (three 96-well plates used in total). All six reagent-only samples produced very low assembled 16S read counts of between 74 to 372 total reads (average 149 reads), indicating negligible contamination of the actual samples which had between 11,929 and 108,169 total reads (average 42,335 reads). ASVs were classified using a pre-trained classifier based on SILVA (release 132), a comprehensive database that provides accurate annotations. ASV counts per sample were exported as biom files from a QIIME2 artifact and converted into a human readable tsv file using “biom convert”. Read counts per sample were rarefied to 11,929 reads per sample (the lowest count among the 242 samples) using the “rrarefy” command in the R package “vegan” (version 2.6-4), and normalized read counts were calculated per sample by dividing the number of reads associated with each ASV by the total number of reads assigned across ASVs. Taxonomic identifications used are directly provided by SILVA (release 132). Raw 16S rRNA can be downloaded from public database (SRA BioProject PRJNA911205; All sample accessions are available in Supplementary Data 2).

For the 16S rRNA/ASV sample analysis, Faith phylogenetic diversity values35 (for α-diversity) and UniFrac38 weighted and unweighted distance, as well as Aitchison distance37 (for β-diversity) were calculated for each sample using QIIME2107. Also for β-diversity, Bray-Curtis distance diversity values were calculated using the “vegdist” function in the “vegan” R library (version 2.6-4).

The correlation between SD and PS was tested using a T-statistic test of the Pearson correlation. The Shapiro-Wilk test for normality was used to determine whether each set of metadata followed a normal distribution prior to performing comparisons of differences of means (all Shapiro-Wilk test results and determinations of normality are provided in Supplementary Data 1C). For data determined to be normally distributed (e.g., mother’s delivery age, healthy eating index, mother’s third trimester TNF-α levels, child birthweight), two-sided T-tests with unequal variance were performed (all T-test statistics and significance values are provided in Supplementary Data 1D). For data that were determined not to be normally distributed using the Shapiro-Wilk test, Mann Whitney U tests (also known as Wilcoxon Rank Sum tests) were used to determine significant differences of mean values (all Mann Whitney U test statistics and significance values are provided in Supplementary Data 1E). Significant differences in categorical variables (i.e., race, sex of child, route of delivery, high-vs-low breast milk feeding) were performed using the Chi-Square test (all Chi-Square test statistics and significance values are provided in Supplementary Data 1F). All of the statistics described in this paragraph were calculated using "Statistics Kingdom" (https://www.statskingdom.com/) (2023 version).

For the differences in β-diversity between and within sample groups (Fig. 4 and Supplementary Figure 3), Benjamini-Hochberg false discovery rate (FDR) correction was performed to correct for multiple testing. FDR correction was not performed for the comparisons of sample metadata presented in Table 1 and Supplementary Data 1A because these were tests performed for an overview of associated metadata prior to testing differences in the GM and were not considered as part of the results. Significant differences in components of SD and PS were expected since samples were chosen from the extremes of phenotype for SD and PS to improve detection of GM differences between groups.

ASV-based sample clustering was performed using non-metric multidimensional scaling (NMDS) using the "metaMDS" in the 'vegan' R package (v 2.6-4), with setting k=2. Dirichlet Multinomial Mixtures (DMM) clustering was performed to separate samples into cluster groups independently of the NMDS plot, using the 'DirichletMultinomial' package (v 1.40.0) in R, using ASVs detected in at least 3 samples. Clustering was performed and tested from n=1 to n=5, and an optimal cluster number per comparison was selected based on minimum log posterior loss correction (lplc) values from DMM, as well as using silhouette scores ("cluster" R package, version 2.1.4) and prediction strength values (“flexible procedures for clustering ; fpc” R package, version 2.2-10), using the NMDS distance matrix and DMM cluster assignments as input.

WMS samples:
The 178 WMS samples were also used as input for HUMAnN3 (version 3), which was run from the biobakery/humann docker container (latest

version as of October 2020) using the Chocophlan nucleotide database and Uniref90 protein database. HUMAnN3 runs the MetaPhlAn (version 3) program as an intermediate step to assign organism-specific functional profiling, and the developer-provided Metaphlan3 bowtie2 database was used for this intermediate step. The HUMAnN3 pipeline was used to generate MetaCyc pathway abundance per sample. The "humann_renorm_table" script (included in the HUMAnN3 distribution) was used to convert Reads Per Kilobase (RPK) values in the MetaCyc abundance table to a normalized value, Copies Per Million (CPM), which can be compared across samples.

For the WMS sequence analysis, samples were divided into "high" and "low" SD and PS based on the distribution of these values across the sample set. Samples above the average value +0.5 standard deviations were considered "high" and samples below the average value -0.5 standard deviations were considered "low" (35 "low-SD", 43 "high-SD, 36 "low-PS" and 32 "high-PS"; Fig. 1C). The same approach was used to separate samples into "high" and "low" sample sets based on inflammatory marker data (IL-6, IL-8, IL-10, and TNFα).

To identify bacterial taxa that strongly predict mothers' SD and PS scores, we analyzed taxonomic and pathway GM profiles using three approaches. First, a supervised machine-learning approach (Random Forest) that identifies non-linear relationships from high dimensional and dependent data was used to (i) quantify the ability to predict metadata classification based on the microbiome profiles, indicative of the overall association between the microbiome and the composite scores, and (ii) for each comparison, identify the specific genomes or pathways that most strongly differentiate between high and low SD and PS scores, ranked based on "mean decrease in accuracy" (MDA; representing the percentage of prediction accuracy that would be lost if a genome/pathway was excluded from the RF training). When discussing RF results, "predictors" is used to indicate the genomes or pathways used in the final RF model (minimum MDA 0.1%). RF was run using the "randomForest" package in R (v4.7-11). The generalization error of the model was evaluated by out of bag (OOB) error. The association of the metadata with the microbiome was quantified using the RF classification accuracy, and the significance of the accuracy was measured using binomial distribution tests (Fig. 5A) with FDR correction applied to correct for the number of tests. RF model accuracy was also examined using receiver operating characteristic (ROC) curves, quantified using the area under the curve (AUC) (Fig. 5B) generated using the R library "ROCR" (version 1.0-11). Significance values for the ROC curves were assigned by Two-sided Mann-Whitney U statistics, using the "roc.area" function in the "verification" R package, version 1.42.

Second, linear discriminant analysis effect size (LEfSe, Galaxy Version 1.0), the most frequently used statistical tool to determine significant differences in microbiome member abundance, was used for differential genome abundance testing (default settings at $P \leq 0.05$ for significance) for the non-parametric factorial Kruskal-Wallis (KW) sum-rank test, and requiring a linear discriminant analysis (LDA) ES (effect size) of at least 2 in order to identify differentially abundant taxa. The same approach was used for the pathway analysis, but the ES test cutoff applied was reduced to a value of 1 instead of 2, since the ES is designed for the more sparse nature of metagenomic abundance data. However, the same RF MDA and LEfSe Kruskal-Wallis cutoffs were applied for pathway analysis. False Discovery Rate (FDR) correction was performed for the LEfSe KW significance values, among the genomes and pathways in the top 25 genomes / pathways identified by the RF analysis (which were the only ones considered for discussion purposes).

Third, ANCOM-BC2, a differential abundance tool for microbiome data which estimates the unknown sampling fractions and corrects the bias induced by their differences among samples, was ran using the "ANCOMBC" R package (version 2.1.4), using genomes detected in at least 3 samples, and the setting prv_cut0.05. This was performed to provide additional confidence in results identified by Random Forest and LEfSe, but was not applied as a filter for identifying taxa of interest to present in figures.

Cytoscape (v 3.10.0) was used to construct networks (Fig. 8) connecting high-SD associated genomes (from Figs. 6 and 7) to high-SD associated pathways (Table 2), with node sizes indicating the mean decrease in accuracy (MDA) values from the random forest analysis, and edge thickness and darkness indicate the proportion of reads that each genome contributes to the pathways (as calculated from the HUMAnN3 output).

For manuscripts utilizing custom algorithms or software that are central to the research but not yet described in published literature, software must be made available to editors and reviewers. We strongly encourage code deposition in a community repository (e.g. GitHub). See the Nature Portfolio guidelines for submitting code & software for further information.

Data

Policy information about availability of data

All manuscripts must include a data availability statement. This statement should provide the following information, where applicable:

- Accession codes, unique identifiers, or web links for publicly available datasets
- A description of any restrictions on data availability
- For clinical datasets or third party data, please ensure that the statement adheres to our policy

The raw 16S sequence reads and whole metagenome shotgun (WMS) sequence reads generated in this study have been deposited in the NCBI Sequence Read Archive (SRA) database under BioProject number PRJNA911205 https://www.ncbi.nlm.nih.gov/bioproject/PRJNA911205. 16S taxonomic identifications were produced using the SILVA database (release 132). WMS sequences were mapped to the Unified Human Gastrointestinal Genome (UHGG) database (version 1), and pathways were quantified from mapping results using HUMAnN3 (version 3), which is based on UniProt/UniRef 2019_01 sequences and annotations. The detailed sample metadata matched to SRA accession numbers, normalized abundance data for all 16S ASVs, WMS genomes and WMS pathways for every sample data are available at in Supplementary Data 2. ASV sequences are available in Supplementary Data 3. Individuals have been de-identified using random identifiers. Individuals have been de-identified using random identifiers. Results from all statistical comparisons are provided in Supplementary Data 1.

Human research participants

Policy information about studies involving human research participants and Sex and Gender in Research.

Reporting on sex and gender	Social disadvantage and psychosocial stress did not vary by sex of the infant, and sex was not considered as a variable of interest in subsequent analysis. Infant sex was determined at the time of birth and sex is indicated per infant participant in Supplementary Table 2. All adults in the study (the mothers of the infants) were self-reported as female. There were no differences reported between assigned sex and gender, so no disaggregation is required. There were no significant differences identified between male and female children in terms of social disadvantage or psychosocial stressors (as shown in table 1), and no distinction between them was used to perform downstream statistical analyses. Sex/gender assignment for each child is provided in Supplementary Table 2.
Population characteristics	The participants in this study are pregnant women and their offspring at age 4 months. Mothers were enrolled in a study of

	Early Life Adversity and Biologic Embedding and Risk for Developmental Precursors of Mental Disorders (ELABE) which began September 1, 2017. Exclusions criteria for the parent study included multiple gestation, congenital infection, and/or alcohol or drug use other than tobacco and marijuana. Subjects were eligible for this study if mothers had delivered at greater than or equal to 37 weeks, her infant had reached 4 mos of life by January 1 2020 (N=355) and she had provided a perinatal stool sample (N=300). Of the 300 mothers with perinatal stools available, 1 mother withdrew, and 149 stool samples were at or post delivery, with 134 mothers with stool sample obtained prenatally (T3). Of these 121 had the infant's 4 month stool samples available. Subject characteristics are outlined in Table 1. Cases and controls were divided into "high' or "low" SD and PS based on distribution of these values across the dataset. Samples above the average value + 0.5 standard deviations were considered "high" and samples below the average value – 0.5 standard deviations were considered "low" (Illustrated in Figure 1C and characteristics evident in Table 1 and Table S1a). The age of the mothers ranged from 19 to 41 years and the age of the children at time of sampling ranged from 4 to 6.9 weeks. Age data is provided for every mother and child in Supplementary Data 2, and age ranges for mothers and children in each comparison are provided in Table 1.
Recruitment	This study draws from the Early Life Adversity and Biological Embedding (eLABE) study, with pregnant women identified from the March of Dimes (MOD) Prematurity Research Center at Washington University in St. Louis. Pregnant women (N=395) were recruited between 2017-2020 and delivered at Barnes Jewish Hospital in St. Louis and were recruited by study personnel during clinic visits. One clinic primarily serves patients with public health insurance, and the other primarily serves patients with private health insurance. Eligibility for MOD enrollment were not restrictive, including plans to deliver at Barnes Jewish Hospital, be 18 years of age or older, and English speaking. Mother. Exclusion criteria included multiple gestations, congenital malformations and infections, premature birth (<37 weeks gestation), maternal alcohol or drug use during pregnancy (excluding tobacco, marijuana), and maternal steroid exposure (excluding inhaled). Race and ethnicity were based on maternal self reporting extracted from the medical record. A priori eligibility for this study at four months included if mothers had delivered at ≥37 weeks and infants had reached 4 months corrected age (N=355), with both maternal 3rd trimester (T3) and infant 4 months stool samples available (Supplementary Fig. 1). Mothers had no record of recent antibiotic usage as of the sampling point at T3, and just four of the children had received antibiotics before the 4-month sampling timepoint (two receiving amoxicillin within the first four weeks, one receiving azithromycin at 26 days, and one continuously receiving PCN-V; Supplementary Data 2). Women facing social disadvantage were oversampled by increasing recruitment for clinics serving low income women, in order to facilitate the intended comparisons based on social disadvantage and psychological stressors. It is possible that the criteria for English speaking only applicants, limits generalizability and increases bias toward a higher Socioeconomic status. Social Disadvantage however was evenly spread across the cohort (Fig 1). The requirement of providing a third trimester stool sample may also bias toward a higher socioeconomic status with resources to retrieve and return samples. We attempted to mitigate this with the use of a courier system that retrieved home produced stool samples for all mothers, at all times, to accommodate variable work hours.
Ethics oversight	All relevant ethical regulations were followed, and the study was approved by the Washington University in St. Louis Institutional Review Board in the Human Research Protection Office (protocol number 201703145), with informed consent obtained from the mother for themselves and their infants. The study was performed in accordance with Strengthening the Reporting of Observational Studies in Epidemiology (STROBE) guidelines. Detailed metadata for all samples are provided in Supplementary Data 2 and are summarized for each comparison in Supplementary Data 1A.

Note that full information on the approval of the study protocol must also be provided in the manuscript.

Field-specific reporting

Please select the one below that is the best fit for your research. If you are not sure, read the appropriate sections before making your selection.

☒ Life sciences ☐ Behavioural & social sciences ☐ Ecological, evolutionary & environmental sciences

For a reference copy of the document with all sections, see nature.com/documents/nr-reporting-summary-flat.pdf

Life sciences study design

All studies must disclose on these points even when the disclosure is negative.

Sample size	Sample size was determined by the availability of a prenatal maternal stool samples and a stool from her infant at 4 months of life. No statistical analysis was used to predetermine sample size, and all available samples meeting metadata criteria were used in the analysis. The total number of biological replicates for the 16S analysis was 121 mother:child pairs (242 total samples) and for the whole metagenome shotgun (WMS) analysis was 89 mother:child pairs (178 total samples).
Data exclusions	One mother sample was excluded when she withdrew from the study a predetermined criteria for inclusion in this study.
Replication	All samples represent unique biological replicates, and the number of replicates used for each test are indicated in the text or in Supplementary Table 1. The total number of biological replicates for the 16S analysis was 121 mother:child pairs (242 total samples) and for the whole metagenome shotgun (WMS) analysis was 89 mother:child pairs (178 total samples). No technical replicates were used in this study. All attempts at replication were successful, and each mother:child sample pair represented independent biological replicates.
Randomization	Allocation to high or low social disadvantage and psychological stress was based on demographic and clinical phenotype and therefore randomization was not applicable.
Blinding	Blinding was done during sample processing and WGS. Blinding was not applicable for subsequent analysis because metadata such as infant age and case/control status was necessary for statistical analysis.

Reporting for specific materials, systems and methods

We require information from authors about some types of materials, experimental systems and methods used in many studies. Here, indicate whether each material, system or method listed is relevant to your study. If you are not sure if a list item applies to your research, read the appropriate section before selecting a response.

Materials & experimental systems

n/a	Involved in the study
☒	☐ Antibodies
☒	☐ Eukaryotic cell lines
☒	☐ Palaeontology and archaeology
☒	☐ Animals and other organisms
☒	☐ Clinical data
☒	☐ Dual use research of concern

Methods

n/a	Involved in the study
☒	☐ ChIP-seq
☒	☐ Flow cytometry
☒	☐ MRI-based neuroimaging
